# Are Ocular and Serum Half-Lives After Ranibizumab Intravitreal Injection Dependent on Dose?

**DOI:** 10.1167/tvst.12.4.9

**Published:** 2023-04-11

**Authors:** Tamara van Donge, Francesco Brizzi, Antonello Caruso, Matthias Fueth, Bernhard Steiert

**Affiliations:** 1 Roche Pharma Research and Early Development, Pharmaceutical Sciences, Roche Innovation Center Basel, F. Hoffmann-La Roche Ltd., Basel, Switzerland. e-mail: tamara.van_donge@roche.com

With great interest we have read the article by Kim et al*.*^1^ entitled “Intraocular Pharmacokinetics of 10-fold Intravitreal Ranibizumab Injection Dose in Rabbits.” The authors concluded that the retinal and serum half-lives (*t*_1/2_) of ranibizumab in rabbit eyes were increased twice after a 10-fold dose compared to the 0.3-mg dose.

Kim et al*.* collected data in 28 rabbits following intravitreal injection of ranibizumab at two dose levels (0.3 and 3.0 mg). The pharmacokinetic data were modeled assuming instantaneous absorption and first-order elimination, resulting in the following monoexponential equation for drug concentration^2^

$$\begin{array}{c} C\left(t\right)\;=\;\frac{Dose}{V/F\;}\times e^{-kt}\; \end{array}$$

where *V*/*F* is the apparent volume of distribution, and the elimination rate constant (*k*) is

$$\begin{array}{c} k\;=\;\;\frac{\ln\left(2\right)}{\;t_{1/2}} \end{array}$$

This model was fitted to each matrix separately to obtain pharmacokinetic parameters (i.e., apparent volume of distribution and apparent clearance) resulting in estimated *t*_1/2_ for each matrix and dose independently. No significant differences were observed between the *t*_1/2_ of the two ranibizumab doses in vitreous (55.58 vs. 53.36 hours) and aqueous (51.70 vs. 52.60 hours) humor. Remarkably, the authors reported a twofold prolongation in retinal and serum *t*_1/2_ values with a 10-fold increase in dose (36.74 vs. 76.85 hours and 91.93 vs. 179.01 hours, respectively). Nevertheless, in the following we indicate several limitations of their estimation and provide a reanalysis of the experimental data, illustrating the instability of *t*_1/2_ estimations.

**Figure 1.**
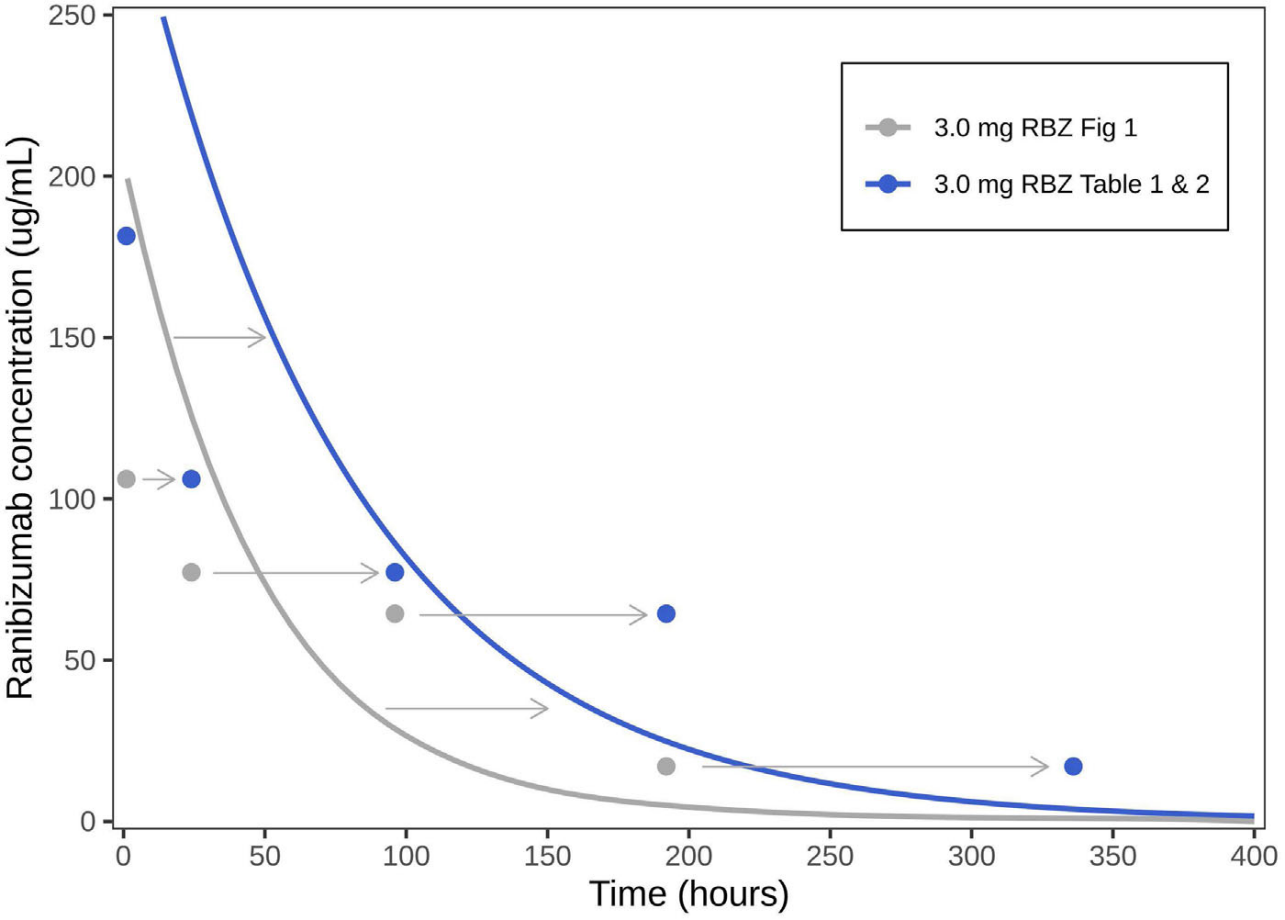
Discordance between the concentration–time data for the aqueous humor observed in Table 1 and Figure 1 of the original paper. *Blue* and *gray* *dots* represent the observations for the 3.0-mg dose as presented in Table 1 and Figure 1 of the original paper, respectively. *Gray* *arrows* denote the differences observed between the data presented in Tables 1 and 2 and Figure 1 of the original paper. The *blue line* represents the simulated concentration–time profile for the aqueous humor when applying the pharmacokinetic parameter values (e.g., *CL*/*F*, *V*/*F*) published in Table 2 of the original paper. The *gray* *line* represents the “predicted trend lines” for aqueous humor as shown in Figure 1 of the original paper. Only data for the 3.0-mg dose are displayed for clarity; the same inconsistencies are observed for the 0.3-mg ranibizumab dose.

**Figure 2.**
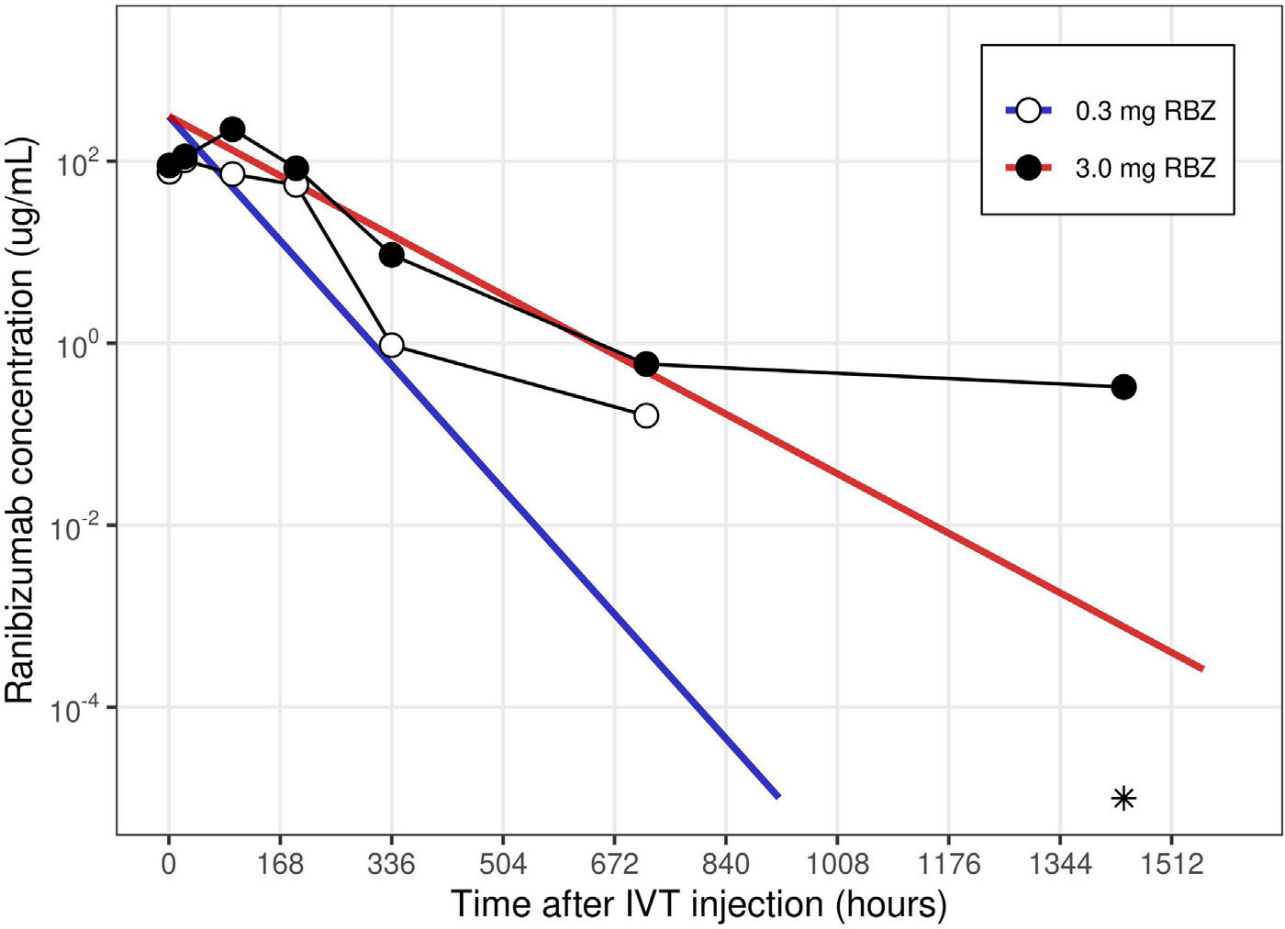
Semilogarithmic plot of retinal concentration–time profiles after intravitreal injection of 0.3 and 3.0 mg ranibizumab. *Symbols* indicate experimental data (mean values) by Kim et al*.*^1^ *Open* and *filled dots* illustrate the mean concentrations for 0.3 and 3.0 mg ranibizumab, respectively. The *asterisk* indicates a zero mean concentration value at 1440 hours for the 0.3-mg group (arbitrarily displayed at 10^–5^ µg/mL). *Blue* and *red lines* represent simulated concentration–time profiles when using pharmacokinetic parameter values (*CL*/*F* and *V*/*F*) published in Table 2 of the original paper for doses of 0.3 and 3.0 mg, respectively.

First, several inconsistencies can be recognized between the pharmacokinetics data reported in Table 1, the data and “predicted trend lines” displayed in Figure 1, and the results of the pharmacokinetics analysis in Table 2 of the original paper. Second, the aqueous humor concentrations shown in Figure 1 of the original paper appear to differ from the data presented in Table 1 of the original paper, as these data points are shifted to the left as illustrated below (Fig. 1). Third, the assumption of monoexponential decline for the retina can be debated. If late data points (low concentrations) are included in the analysis, a biexponential decline may be recognized (Fig. 2). Although the parameter estimation heavily relies on inclusion or exclusion of suspect last data points, the original paper does not provide any detail on the handling of such points. Similarly, the assumption of an instantaneous absorption does not seem to hold, as an absorption phase is present in the retina and serum (Fig. 1, original paper) that should be excluded in the estimation of the terminal elimination rate.^3^ Furthermore, the principle of flip-flop kinetics is a well-known phenomenon after intravitreal administration that results in similar half-lives in the ocular matrices and in serum, although this is not recognized in the published analysis.^4^ Fourth, information on the lower quantification limit and on the handling of the data below this limit is lacking. For the assumed first-order elimination kinetics, the estimation of *t*_1/2_ is mainly driven by the last data points, characterized by low concentrations. Were some concentration data below the limit of quantification? If so, were these values set to zero, discarded, or divided by two for the pharmacokinetic analyses?^5^ The estimated *t*_1/2_ depends on the answers to these questions. Fifth, the small sample size of the experiment only allows reliable estimation of the elimination rate constant (and *t*_1/2_). It is difficult to accurately estimate the apparent clearance and volume parameters, and we expect that these estimates have high associated uncertainty, despite confidence intervals are not reported in Table 2 of the original paper.

**Table 1.** Half-Life Estimates for Each Matrix and Dose Based on a Noncompartmental Analysis

	0.3 mg Ranibizumab			3.0 mg Ranibizumab		
Matrix	Half-Life (h) (95% CI)	Included Time Points (h)	Adjusted *R*^2^	Half-Life (h) (95% CI)	Included Time Points (h)	Adjusted *R*^2^
Vitreous	51.7 (27.1–568)	96, 192, 336	0.99	52.6 (19.5–∞)	192, 336, 720	0.96
Aqueous	57.5 (14.3–∞)	192, 336, 720	0.89	96.5 (68.7–162)	24, 96, 192, 336, 720, 1440	0.90
Retina	67.8 (30.2–∞)	96, 192, 336, 720	0.79	173 (63.8–∞)	192, 336, 720, 1440	0.64
				77.6 (22.5–∞)^*^	192, 336, 720^*^	0.93^*^
Serum	955(105–∞)	192, 336, 720	0.43	1102 (489–∞)	336, 720, 1440	0.98

* Results of the noncompartmental analysis for the retina after exclusion of the last data point (1440 hours).

To address the issue of model selection, the concentration data in the original Table 1 were reanalyzed, and the half-life in each matrix was estimated by fitting the terminal phase to an exponential function (noncompartmental analysis was performed in R 3.6.1 with RStudio 1.2.5001 (R Foundation for Statistical Computing, Vienna, Austria) (Table 1).^6^^,^^7^ In the retina, the *t*_1/2_ value of 67.8 hours obtained for the low dose is comparable to the estimate for the high dose (77.6 hours) when excluding the last data point (1440 hours) (Table 1). The 95% confidence intervals for both doses are overlapping and very wide, highlighting substantial uncertainty due to the small sample size. Also, the retina *t*_1/2_ estimates appear to be highly variable depending on which terminal data points are included in the analysis; for example, if the last data point were included then the retinal half-life would be estimated to be 173 hours, with wide confidence intervals. Similar considerations can be made regarding the serum data and estimates.

Therefore, after identifying several inconsistencies in the original paper by Kim et al*.*^1^ and reanalyzing the experimental data, we were unable to find supporting evidence for different retinal and serum half-lives for the two investigated doses. Most importantly, given the limited data points, the inherent variation in biological data, and the lack of details on the quantification limits, the pharmacokinetic parameter estimates will remain controversial and highly uncertain, irrespective of the analysis method used and the points that are included. In closing, the study by Kim et al*.*^1^ provides no conclusive evidence for a dependence of retinal and serum half-life on dose.
